# Grade III Ductal Carcinoma In Situ Arising Within a Long-Standing Fibroadenoma: A Case Report Illustrating the Limits of Shape-Based Breast Imaging Reporting and Data System (BI-RADS) and AI-Assisted Ultrasound Assessment

**DOI:** 10.7759/cureus.117009

**Published:** 2026-09-27

**Authors:** Abdulkadir Eren, Emrah Karatay, Can Yilmaz Yozgat

**Affiliations:** 1 Radiology, Istanbul Medipol University Mega Hospital, Istanbul, TUR; 2 Radiology, Sultan II. Abdulhamid Han Training and Research Hospital, Istanbul, TUR; 3 Emergency Medicine, Hatay Kirikhan State Hospital, Hatay, TUR

**Keywords:** artificial intelligence, bi-rads, breast ultrasound, computer-aided diagnosis, ductal carcinoma in situ, fibroadenoma, pet/ct, shear-wave elastography

## Abstract

A 40-year-old woman with no personal or family history of breast cancer had a right breast mass first characterized as a probable fibroadenoma on an outside MRI evaluation. A repeat MRI performed years later again described a well-circumscribed, benign-pattern (type 1) enhancing nodule assessed as Breast Imaging Reporting and Data System (BI-RADS) 3. Targeted ultrasound showed an oval, parallel-oriented, circumscribed hypoechoic mass, and an integrated artificial intelligence (AI) lesion-characterization tool returned a categorical output of benign (BI-RADS 2) based on the shape and orientation descriptors displayed on screen. However, shear-wave elastography of the same lesion showed markedly elevated stiffness (maximum elasticity 135.48 kPa), prompting the radiologist to revise the overall assessment to BI-RADS 4 and pursue ultrasound-guided tru-cut biopsy, which revealed solid, cribriform, and comedo-type high-grade (nuclear grade 3) ductal carcinoma in situ (DCIS) arising within a fibroadenoma. Whole-body 18F-fluorodeoxyglucose (18F-FDG) PET/CT subsequently showed FDG uptake at the known primary site with no other avid disease, while histopathology established the diagnosis and stage. The patient underwent segmental mastectomy with sentinel lymph node biopsy; final pathology confirmed a 1.5 × 1 cm focus of high-grade DCIS entirely confined to the fibroadenoma, with clear surgical margins and no nodal involvement (pTisN0(i-,sn)). This case demonstrates that malignancy, including high-grade DCIS, can arise within an otherwise typical, long-stable fibroadenoma and can retain a benign morphological profile on B-mode ultrasound and AI-assisted shape-based classification. Quantitative elastographic stiffness discordant with a benign shape classification should not be dismissed and warrants biopsy. Multiparametric correlation, combining morphologic assessment with quantitative elastography, remains essential and should not be superseded by a single shape-based descriptor, human or algorithmic. Ultimately, in this case, only tissue sampling provided a clear and definitive diagnosis, underscoring that biopsy should not be deferred when a breast mass is reassessed after a long interval or shows any feature discordant with a straightforward fibroadenoma.

## Introduction

Fibroadenoma is the most common benign solid mass of the female breast, typically occurring in women under 35 years of age and characterized sonographically by an oval or gently lobulated shape, circumscribed margins, parallel orientation, and homogeneous hypoechogenicity, the constellation of features that anchors the Breast Imaging Reporting and Data System (BI-RADS) 3 (“probably benign”) category. The coexistence of carcinoma within a fibroadenoma is sometimes loosely termed “malignant transformation,” although this case does not establish a proven biological transformation of the fibroadenoma itself; it is a well-described but rare phenomenon: pooled series place the incidence between roughly 0.002% and 0.125% of excised fibroadenomas, with carcinoma in situ, predominantly lobular, less often ductal, outnumbering invasive carcinoma within this subgroup [1-3]. The reported mean age at diagnosis (42-47 years) is notably older than the typical age of fibroadenoma presentation, suggesting that longstanding lesions under long-interval follow-up are a particular context in which this can occur [3-5].

Because these tumors so often retain the benign morphology of their host fibroadenoma, they are notoriously difficult to distinguish from ordinary fibroadenomas on conventional imaging, and the diagnosis is usually made only after biopsy or excision [6]. The increasing use of quantitative elastography and artificial intelligence (AI)-based lesion characterization software in everyday ultrasound practice adds a further layer to this problem. In the present case, the embedded AI tool (Breast Assistant, powered by Koios DS™ (Koios DS Breast); Koios Medical, Inc., New York, NY, USA), supplied as an integrated component of the hospital’s GE LOGIQ E10 ultrasound platform (GE HealthCare, Chicago, IL, USA), generated a categorical benign/BI-RADS output from the gray-scale image with a manually selected region of interest; the tool does not ingest elastographic data, which was acquired as an independent examination. Detailed software build/version and country-specific regulatory clearance status were not available to the authors beyond the manufacturer’s standard product designation. This should be regarded as a single discordant/false-negative output in this individual case rather than evidence of a general algorithmic mechanism, since publicly available device information does not establish that the tool’s overall risk output depends solely on the displayed shape and orientation descriptors [7-9]. We report a case of high-grade ductal carcinoma in situ (DCIS) arising within a fibroadenoma that had been radiologically stable and repeatedly labeled as probably benign for six years, in which an AI-assisted ultrasound tool classified the lesion as benign despite a markedly elevated elastographic stiffness value, to highlight this diagnostic pitfall and the imaging clues that ultimately prompted biopsy.

## Case presentation

A 40-year-old woman with no personal or family history of breast cancer and no regular medication use presented for re-evaluation of a known right breast mass. An outside breast MRI performed at initial imaging had described a 12 × 8 mm, well-circumscribed, gently lobulated mass in the upper-inner right breast, posterior in location, with type 1 (persistent, benign-pattern) post-contrast enhancement, interpreted as a probable fibroadenoma; the lesion was followed clinically without biopsy. The original MRI report from this outside institution was reviewed by the authors; the original MRI images were not retrievable for inclusion, as the study had been performed at a different institution.

Six years later, a repeat breast MRI at an outside institution again demonstrated the lesion, now measuring 11 × 8 mm, located at the 1 o’clock position approximately 7 cm from the nipple. The mass remained well-circumscribed with a T2-hyperintense signal and type 1 post-contrast kinetics and was again assessed as probably a fibroadenoma (BI-RADS 3) (Figure 1).

**Figure 1:**
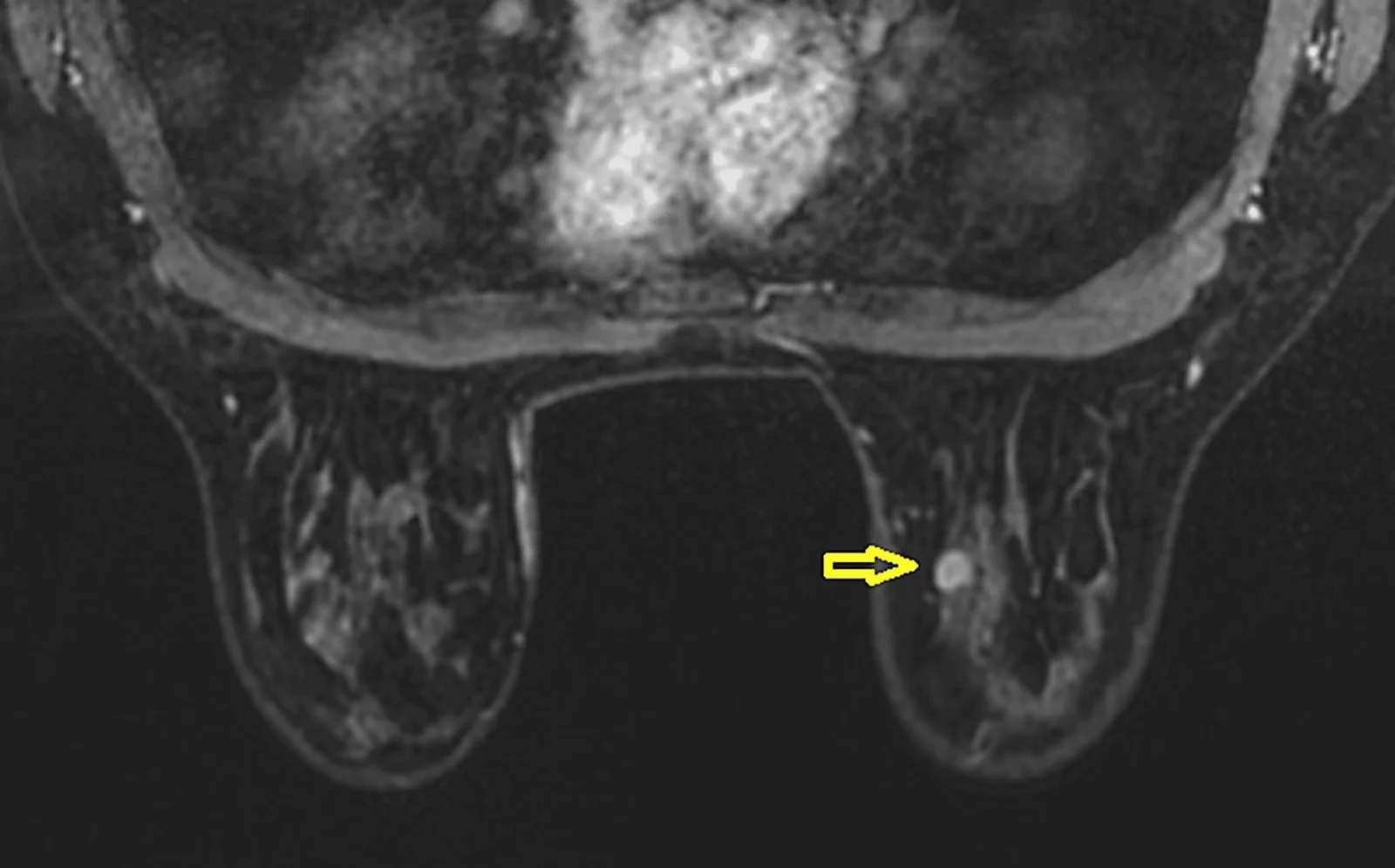
Axial contrast-enhanced MRI of both breasts Showing a well-circumscribed, benign-appearing enhancing nodule in the right breast (arrow), corresponding to the lesion followed serially over the preceding six years.

No intervening imaging was documented in the accessible records between the two MRI examinations. Only the report of this six-year follow-up MRI, accessed through the national e-Nabız health records system, was available to the authors; the original images could not be retrieved, although the reported lesion dimensions were essentially unchanged from the initial study (12 × 8 mm vs. 11 × 8 mm). Given the interval evaluation, the patient was referred for targeted ultrasound.

Diagnostic mammography, performed at an outside institution at the time of re-evaluation without digital breast tomosynthesis, showed heterogeneously dense breast tissue (BI-RADS density category C), with a subtle asymmetric opacity corresponding to the known lesion in the medial portion of the right breast on the craniocaudal view, without associated suspicious microcalcifications; the final mammographic assessment was BI-RADS 2 (Figure 2).

**Figure 2:**
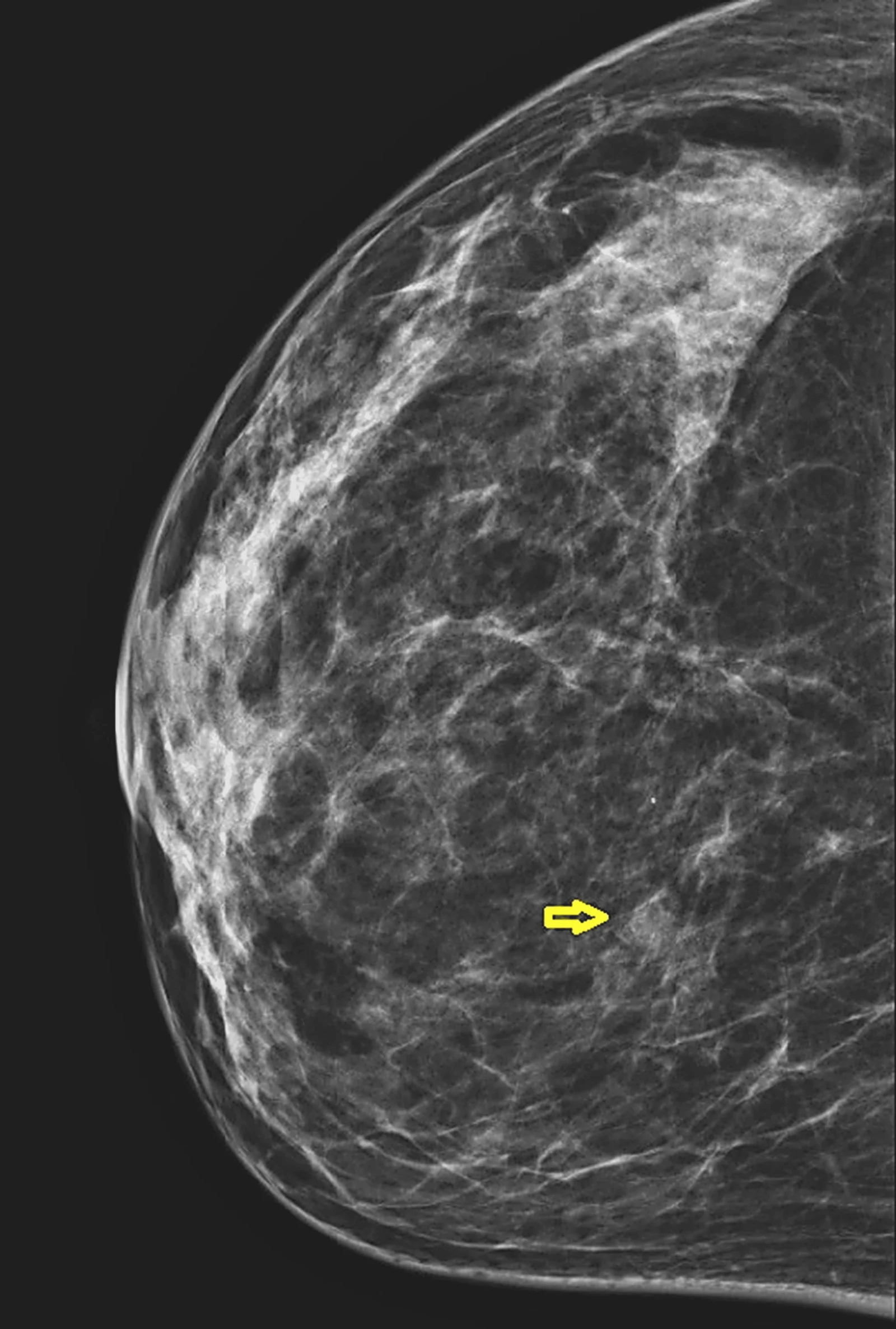
Diagnostic mammogram (craniocaudal view) of the right breast in a heterogeneously dense breast (BI-RADS density category C) A subtle asymmetric opacity, assessed as BI-RADS 2, is present at the site of the known lesion (arrow), partially obscured by the surrounding dense fibroglandular tissue. BI-RADS: Breast Imaging Reporting and Data System

The mammographic appearance did not independently prompt biopsy; the decision to biopsy was driven primarily by the elastographic finding described below.

Gray-scale ultrasound, performed using a GE Healthcare LOGIQ E10 system, confirmed an oval, circumscribed, parallel-oriented hypoechoic solid mass at the 12-1 o’clock position of the right breast, running parallel to the skin surface, a morphology entirely consistent with fibroadenoma, measuring 0.91 × 0.62 × 0.84 cm (volume 0.25 mL) on standard calipers (Figure 3).

**Figure 3:**
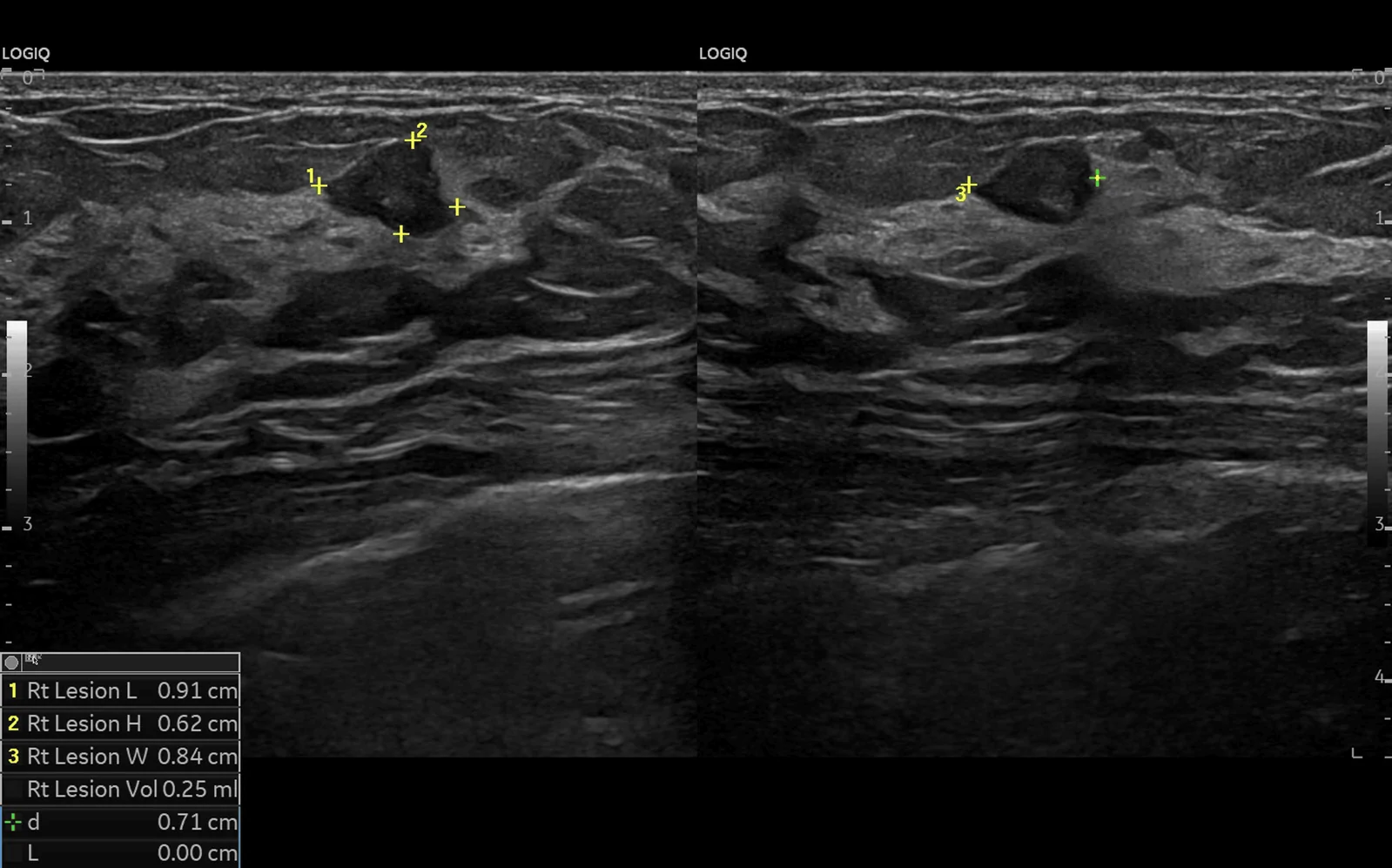
Dual-panel gray-scale ultrasound of the right breast lesion with standard calipers (date/time stamp removed) The mass measured 0.91 × 0.62 × 0.84 cm (volume 0.25 mL) and was oval, circumscribed, and parallel-oriented, a morphology indistinguishable from a simple fibroadenoma.

An integrated AI-based lesion-characterization tool (Breast Assistant, powered by Koios DS™), embedded in the LOGIQ E10 platform, displayed shape and orientation descriptors alongside a categorical output of “Benign, Risk Alignment BI-RADS 2” (Figure 4), reinforcing the reassuring morphological impression; as noted below, the tool’s full underlying decision logic is proprietary and not limited to the descriptors shown on screen.

**Figure 4:**
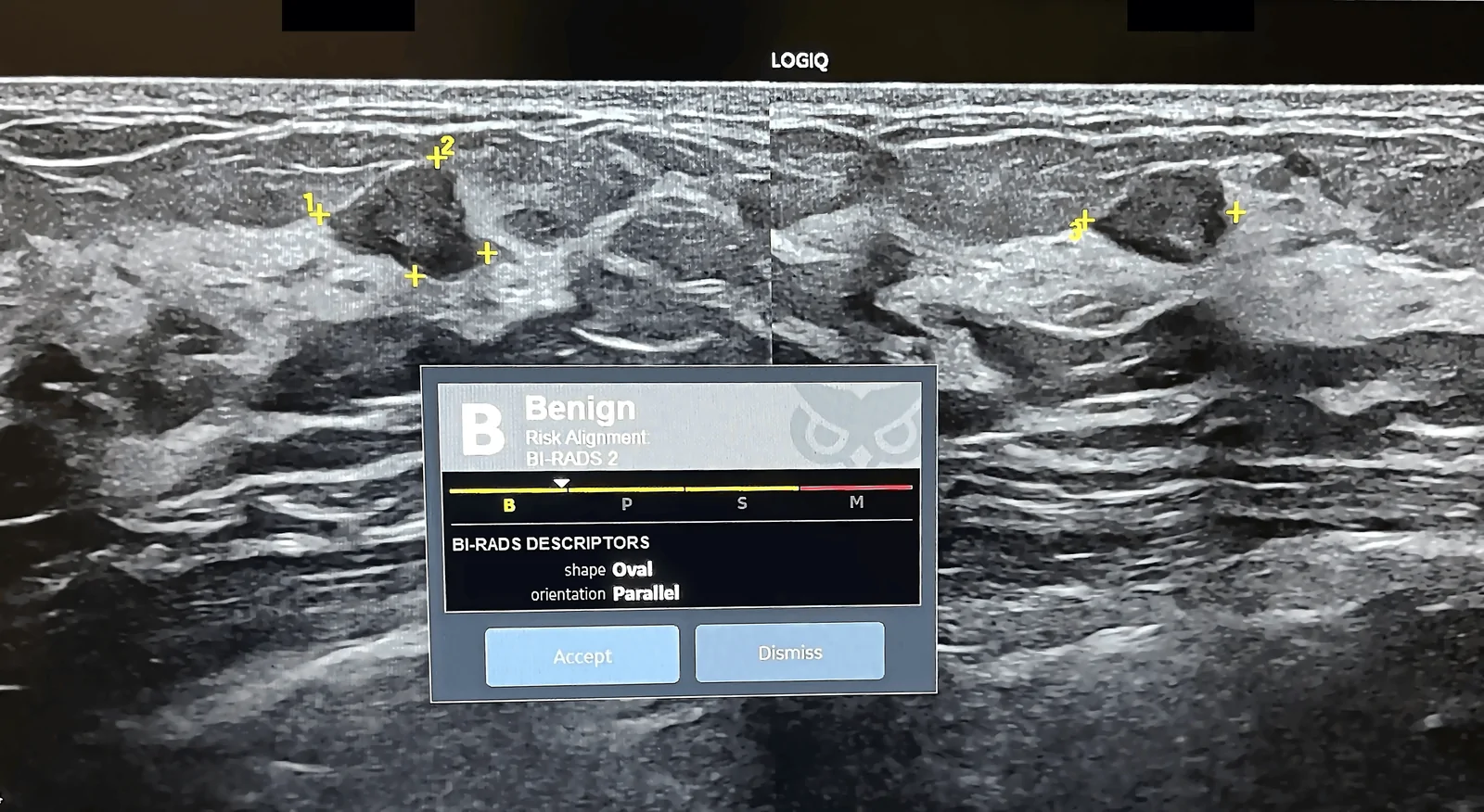
Gray-scale ultrasound with an integrated AI-based lesion-characterization overlay (date/time stamp removed) The displayed shape (“oval”) and orientation (“parallel”) descriptors accompanied the software’s categorical output of “Benign” (BI-RADS 2), which was discordant with the eventual histopathological diagnosis of high-grade DCIS. BI-RADS: Breast Imaging Reporting and Data System; DCIS: ductal carcinoma in situ

However, shear-wave and strain elastography of the same lesion told a different story. Elastography was performed on the same GE LOGIQ E10 platform with the patient supine and the ipsilateral arm raised; a single region of interest (Q-box) was manually placed to fit within the lesion at its area of greatest stiffness (the reddest color-coded region). The maximum elasticity value (E-max) within the lesion measured 135.48 kPa on this single measurement (Figure 5); repeat acquisitions, a formal quality/reliability map, and Emean/standard deviation were not systematically recorded, which we acknowledge as a limitation, since a single maximum-value measurement is more susceptible to boundary or pressure artifact than an averaged multi-acquisition protocol.

**Figure 5:**
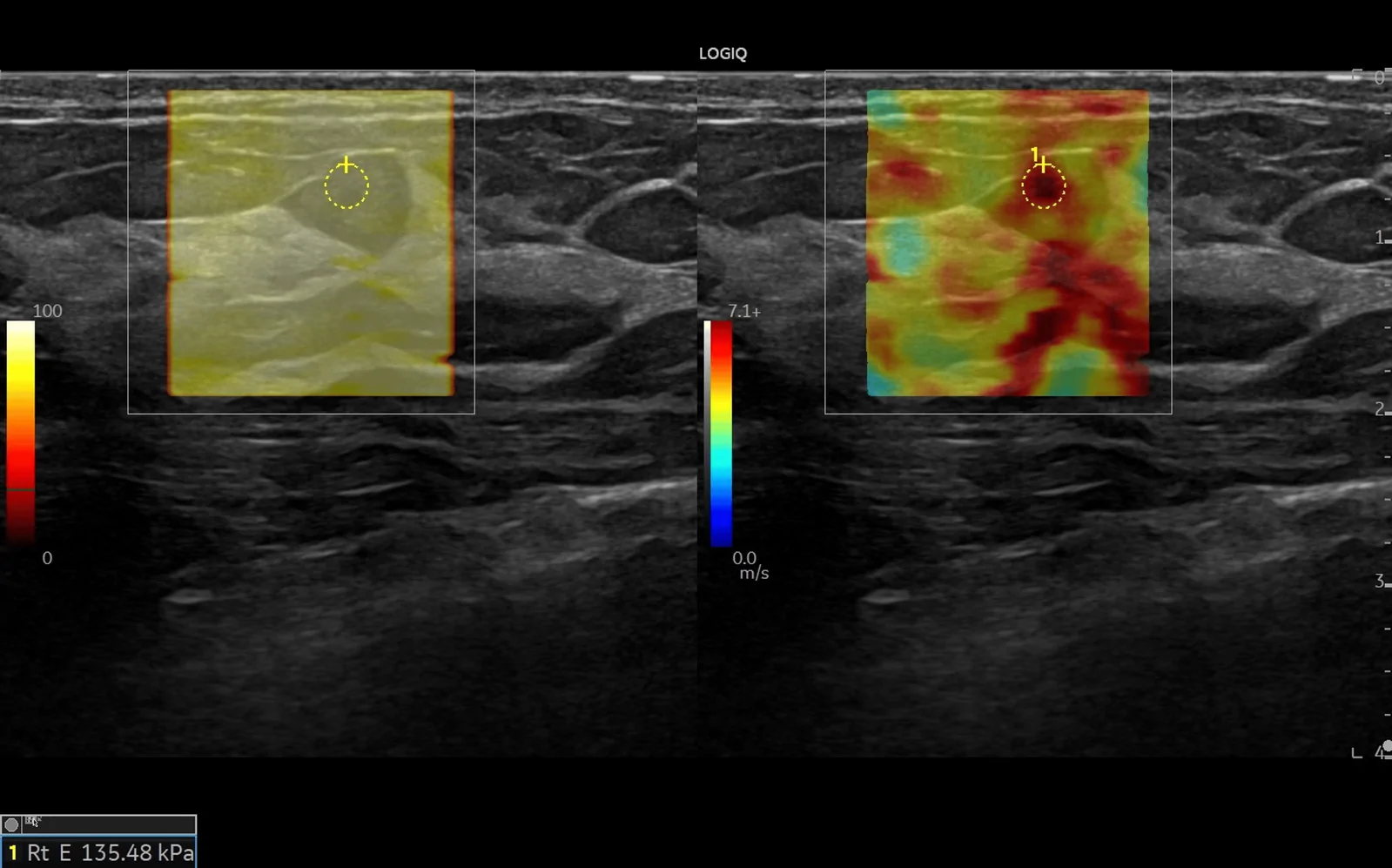
Dual-panel elastography of the right breast lesion (date/time stamp removed) Left panel: strain elastography showing a predominantly stiff (yellow-white) region of interest; right panel: color-coded shear-wave elastogram of the same lesion with extensive red (high-stiffness) areas; the corresponding quantitative maximum elasticity value, from a single measurement, was 135.48 kPa, exceeding thresholds reported in several published shear-wave elastography series (see Discussion for acquisition details and caveats).

This value nonetheless exceeded thresholds reported in several elastography series, which cluster around roughly 30-80 kPa depending on the elastographic system, region-of-interest selection, and reference population used, although no single stiffness cut-off is universally validated across platforms [10-13]. The corresponding strain elastogram likewise showed a large area of red (hard) color coding involving most of the region of interest, with only a peripheral rim of softer (green/yellow) tissue: a pattern more typical of a stiff, potentially malignant process than of a simple fibroadenoma.

Given the discordance between the shape-based benign classification and the markedly elevated quantitative stiffness, the overall sonographic assessment was revised by the requesting radiologist to BI-RADS 4, superseding the AI tool’s BI-RADS 2 output, and ultrasound-guided tru-cut biopsy of the lesion was performed after appropriate preparation and local anesthesia, without immediate complication. Histopathological and immunohistochemical examination (E-cadherin diffusely positive; p63 and CK5/6 positive in myoepithelial cells only; estrogen receptor strongly and diffusely positive in 100% of the in situ tumor cell nuclei) established the diagnosis of solid, cribriform, and comedo-type ductal carcinoma in situ arising within a fibroadenoma (ICD-O 8500/2). Nuclear grade was reported by the pathology laboratory as Grade III on a three-tier (I-III) system, corresponding to high nuclear grade (grade 3 of 3).

Given the high nuclear grade of the DCIS component and residual concern for an occult invasive focus not sampled on core biopsy, whole-body 18F-fluorodeoxyglucose (FDG) PET/CT was performed at the treating team’s discretion, following the department’s standard clinical protocol (patient fasting, confirmation of blood glucose within an acceptable range, and weight-based intravenous 18F-FDG dosing with imaging acquired after a standard post-injection uptake interval); PET/CT is not part of routine staging for biopsy-proven pure DCIS. Granular acquisition parameters (exact injected activity, blood glucose value, uptake time, scanner model/reconstruction, and lesion SUVmax) were not separately extracted for this retrospective case report, which we acknowledge as a limitation. Imaging demonstrated a single focus of pathological FDG uptake corresponding to the primary lesion in the upper right breast (Figure 6, arrow), with no FDG-avid axillary, regional, or distant disease to suggest nodal or metastatic spread.

**Figure 6:**
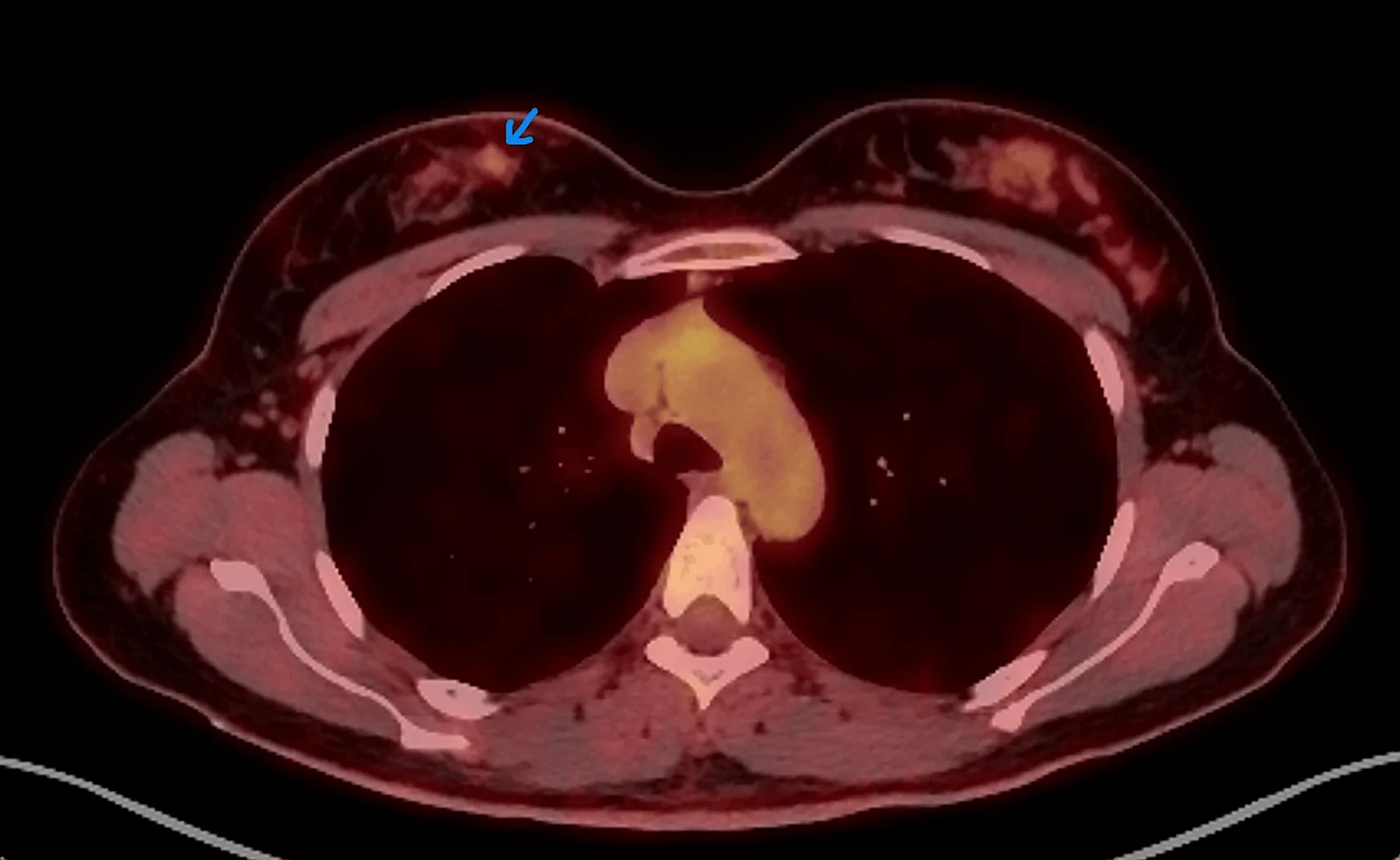
Axial fused 18F-FDG PET/CT image of the chest at the level of the breasts, obtained for pre-treatment staging The arrow indicates focal pathological tracer uptake corresponding to the known right breast primary; no FDG-avid axillary, internal mammary, or distant disease was identified.

This finding helped exclude regional and distant disease prior to surgery; eligibility for breast-conserving management was determined on the basis of tumor size, breast-to-lesion ratio, and the absence of invasion on core biopsy, rather than by the PET/CT result itself.

Given the high nuclear grade of the DCIS component, its size, and the possibility of an occult invasive focus not excluded by core biopsy, the patient subsequently underwent segmental mastectomy of the right breast together with sentinel lymph node biopsy, consistent with recommendations to consider sentinel node sampling in patients with DCIS undergoing mastectomy or when invasion cannot be confidently excluded. Intraoperative frozen-section analysis of the sentinel nodes was negative for malignancy in all three nodes examined, and a frozen section of the segmental mastectomy specimen confirmed DCIS confined to the area of the known lesion with no evidence of invasion. On final pathology, the excised specimen contained a fibroadenoma harboring comedo, solid, and cribriform-type high-grade DCIS measuring 1.5 × 1 cm in situ, with associated microcalcification and without dermal or skin involvement. Surgical margins were clear, with the nearest (superior) margin 1.5 cm from the tumor. The surrounding breast parenchyma showed sclerosing adenosis, adenosis, microcalcification, and cystic papillary apocrine metaplasia. All three sentinel lymph nodes were free of metastatic disease. The final pathological stage was pTisN0(i-,sn), and the patient was referred for adjuvant multidisciplinary discussion.

## Discussion

This case illustrates two closely related teaching points relevant to contemporary breast imaging practice. First, it reinforces that carcinoma, including clinically significant, high-grade DCIS, can arise within a fibroadenoma that has been radiologically stable and repeatedly labeled “probably benign” over a period of years. Breast carcinoma arising within a fibroadenoma remains rare, with reported incidences well under 1% of excised fibroadenomas, and in situ disease (particularly lobular carcinoma in situ) more common than invasive carcinoma within this subgroup [1-4]. The peak age of occurrence in the mid-40s, together with case reports describing carcinoma identified years into imaging follow-up of a lesion originally interpreted as fibroadenoma, indicates that long-term morphological stability alone cannot fully exclude clinically significant disease once a patient has moved beyond the typical fibroadenoma age range; this reflects the identification of carcinoma within a lesion radiologically interpreted as fibroadenoma, rather than a proven biological transformation of the fibroadenoma itself, and the absolute incidence of this occurrence remains very low [5]. In the present case, the lesion had been imaged and labeled as a probable fibroadenoma across a six-year interval before biopsy was pursued.

Second, and more novel from a technical standpoint, this case highlights a specific pitfall of shape-based lesion characterization, whether performed by a human reader applying the BI-RADS lexicon or by an integrated AI-based decision-support tool. Because the malignant component in this case developed within, and largely retained the macroscopic contour of, a pre-existing fibroadenoma, both gray-scale morphology and the embedded AI-based classification tool returned a reassuring “benign” output; put plainly, the displayed AI output was falsely reassuring in this individual case. The tool’s full internal decision logic and relative feature weighting are proprietary and cannot be inferred from a single case, but this observation is consistent with broader reports that computer-aided diagnosis systems can be misled when a malignant process does not produce the expected irregular, non-parallel, angular appearance [7-9]. In this case, quantitative shear-wave elastography provided the discordant signal that morphology alone could not: an E-max of 135.48 kPa exceeded thresholds reported in several elastography series, which cluster around roughly 30-80 kPa depending on the elastographic system, region-of-interest selection, and reference population used, although no single stiffness cut-off is universally validated across platforms [10-13]. When elastography is technically adequate and materially discordant with B-mode morphology, as in this case, the discrepancy should be resolved by repeat assessment and, where clinically appropriate, tissue sampling; here, this discordance was the key finding that prompted biopsy rather than continued surveillance. Mechanistically, this concealment most plausibly reflects the pattern of tumor growth rather than any failure of image interpretation per se: the DCIS component appears to have expanded within the pre-existing fibroadenoma without breaching its stromal pseudocapsule or adopting an infiltrative, spiculated growth pattern, so the lesion's external contour, margin, and orientation, the very descriptors that anchor a "probably benign" classification, whether assigned by a radiologist or an algorithm, were substantially preserved even as high-grade malignancy developed internally. This is consistent with other reported cases of carcinoma arising within fibroadenoma, in which in situ or early disease is often entirely contained by the fibroadenoma's own capsule and is therefore recognizable only on tissue sampling, not on shape-based imaging criteria [3,5].

In practical terms, this means that no degree of shape-based reassurance, human or algorithmic, can substitute for tissue diagnosis once a lesion is reassessed after a long interval or shows any discordant feature; excisional or core biopsy remains the only method that resolves this diagnostic uncertainty with certainty, a point developed further below. The modest variation in lesion size and location across modalities in this case (MRI: 11-12 × 8 mm at the 1 o’clock position; ultrasound: 0.91 × 0.62 × 0.84 cm at the 12-1 o’clock position; final histological DCIS focus: 1.5 × 1 cm) is consistent with the expected differences between imaging-based measurement of the enhancing or sonographically visible component and the full histological extent of disease, together with modality-specific differences in measurement technique and resolution.

The role of 18F-FDG PET/CT in this case was to exclude regional and distant disease prior to definitive surgery in a diagnostically atypical, high nuclear-grade lesion; it did not itself alter the surgical plan and is presented here as a staging adjunct used in this individual case rather than as a routine step or a central teaching point of the report. The absence of axillary or distant FDG-avid disease was consistent with the pathologically confirmed pTisN0(i-,sn) stage; PET/CT is not part of routine staging for pure DCIS and was used selectively here given the residual preoperative concern for an occult invasive focus.

From a management perspective, this case followed a conventional pathway for high-grade DCIS, segmental (breast-conserving) mastectomy with sentinel lymph node biopsy, which is broadly consistent with reported management patterns for carcinoma arising within fibroadenoma, where breast-conserving surgery has been used in the majority of reported cases, reserving mastectomy or axillary dissection for cases with more extensive or invasive disease [1,2]. The clear margins and node-negative status achieved here reflect the value of adequate surgical clearance around the entire fibroadenoma, not merely the biopsy-proven focus, given the propensity of these lesions to extend to or beyond the edge of the benign tumor.

This case should encourage a degree of caution when a stable, benign-appearing breast mass is re-imaged after a long interval and when quantitative elastographic or other functional parameters are discordant with a reassuring shape-based classification, whether that classification is generated by a radiologist or by AI-assisted software. In such circumstances, tissue sampling should be favored over continued morphology-based surveillance, and AI-based BI-RADS tools are best used as an adjunct to, rather than a replacement for, full multiparametric assessment. We acknowledge as a limitation that, after six years of apparent radiological stability, current guidance would often support downgrading such a lesion to BI-RADS 2; the persistence of a BI-RADS 3 assessment at the outside institution may reflect a conservative, institution-specific practice pattern; as noted above, only the report of the six-year follow-up MRI was accessible to the authors, precluding independent image-based comparison, although the reported dimensions indicated essentially unchanged lesion size. This case should not be read as an argument for biopsying every long-stable, imaging-typical fibroadenoma: the absolute incidence of malignancy in this setting is very low, and unnecessary biopsy carries its own costs, discomfort, and occasional complications; rather, it argues for a low threshold to reassess, and where appropriate, sample, specifically when a mass is reassessed after a long interval or shows a discordant feature such as markedly elevated stiffness. Put plainly, imaging morphology and AI-based shape classification could not resolve this lesion, and only histological sampling did; for any breast mass whose behavior, duration of follow-up, or ancillary findings depart from the straightforward, short-interval fibroadenoma picture, excisional or core biopsy provides a clear and definitive diagnosis and should not be withheld in favor of further imaging alone.

## Conclusions

High-grade (nuclear grade 3) DCIS can arise within an otherwise typical fibroadenoma and can remain sonographically and radiologically indistinguishable from a benign lesion, even after years of stable follow-up. In this case, both gray-scale morphology and an AI-assisted shape-based classifier returned a reassuring benign categorization, and only quantitative shear-wave elastography raised suspicion before tissue diagnosis was pursued; as a single case, this cannot by itself establish elastography or AI-assisted tools as reliable substitutes for biopsy in this setting. Rather, the clearest and most generalizable lesson from this case is a simple one: when a breast mass is reassessed after a long interval or shows any feature discordant with a straightforward, short-interval fibroadenoma, tissue sampling by core or excisional biopsy provides a clear and definitive diagnosis and should not be deferred in favor of continued shape-based surveillance, whether that surveillance is guided by a radiologist or by AI-assisted software.
