# Correction: Grade III Ductal Carcinoma In Situ Arising Within a Long-Standing Fibroadenoma: A Case Report Illustrating the Limits of Shape-Based Breast Imaging Reporting and Data System (BI-RADS) and AI-Assisted Ultrasound Assessment

**DOI:** 10.7759/cureus.c489

**Published:** 2026-10-09

**Authors:** Abdulkadir Eren, Emrah Karatay, Can Yilmaz Yozgat

**Affiliations:** 1 Radiology, Istanbul Medipol University Mega Hospital, Istanbul, TUR; 2 Radiology, Sultan II. Abdulhamid Han Training and Research Hospital, Istanbul, TUR; 3 Emergency Medicine, Hatay Kirikhan State Hospital, Hatay, TUR

This article has been corrected as follows: Figures 1 and 2 titles have been corrected for accuracy and two minor typos in the MRI description and mammography description have also been corrected. These corrections affect only the figure legends and two descriptive phrases; the figures themselves, the findings, and the conclusions of the report are unchanged.

